# *In vivo* PET imaging of beta-amyloid deposition in mouse models of Alzheimer's disease with a high specific activity PET imaging agent [^18^F]flutemetamol

**DOI:** 10.1186/s13550-014-0037-3

**Published:** 2014-08-01

**Authors:** Anniina Snellman, Johanna Rokka, Francisco R López-Picón, Olli Eskola, Mario Salmona, Gianluigi Forloni, Mika Scheinin, Olof Solin, Juha O Rinne, Merja Haaparanta-Solin

**Affiliations:** 1 Medicity/PET Preclinical Laboratory, Turku PET Centre, University of Turku, Tykistökatu 6 A, Turku 20520, Finland; 2 Radiopharmaceutical Chemistry Laboratory, Turku PET Centre, University of Turku, Porthaninkatu 3, Turku 20500, Finland; 3 Mario Negri Institute for Pharmacological Research, Milan 20156, Italy; 4 Department of Pharmacology, Drug Development and Therapeutics, University of Turku, Kiinamyllynkatu 10, Turku 20520, Finland; 5 Unit of Clinical Pharmacology, TYKSLAB, Turku University Hospital, Kiinamyllynkatu 10, Turku 20520, Finland; 6 Turku PET Centre, University of Turku, Turku 20521, Finland

**Keywords:** Alzheimer's disease, [18F]flutemetamol, Preclinical evaluation, Beta-amyloid, Transgenic mouse model, High specific activity, APP23, Tg2576, APPswe-PS1dE9, Amyloid imaging

## Abstract

**Background:**

The purpose of the study was to evaluate the applicability of ^**18**^F-labelled amyloid imaging positron emission tomography (PET) agent [^**18**^F]flutemetamol to detect changes in brain beta-amyloid (Aβ) deposition *in vivo* in APP23, Tg2576 and APPswe-PS1dE9 mouse models of Alzheimer's disease. We expected that the high specific activity of [^**18**^F]flutemetamol would make it an attractive small animal Aβ imaging agent.

**Methods:**

[^**18**^F]flutemetamol uptake in the mouse brain was evaluated *in vivo* at 9 to 22 months of age with an Inveon Multimodality PET/CT camera (Siemens Medical Solutions USA, Knoxville, TN, USA). Retention in the frontal cortex (FC) was evaluated by Logan distribution volume ratios (DVR) and FC/cerebellum (CB) ratios during the late washout phase (50 to 60 min). [^**18**^F]flutemetamol binding to Aβ was also evaluated in brain slices by *in vitro* and *ex vivo* autoradiography. The amount of Aβ in the brain slices was determined with Thioflavin S and anti-Aβ_1−40_ immunohistochemistry.

**Results:**

In APP23 mice, [^**18**^F]flutemetamol retention in the FC increased from 9 to 18 months. In younger mice, DVR and FC/CB_50-60_ were 0.88 (0.81) and 0.88 (0.89) at 9 months (*N* = 2), and 0.98 (0.93) at 12 months (*N* = 1), respectively. In older mice, DVR and FC/CB_50-60_ were 1.16 (1.15) at 15 months (*N* = 1), 1.13 (1.16) and 1.35 (1.35) at 18 months (*N* = 2), and 1.05 (1.31) at 21 months (*N* = 1). In Tg2576 mice, DVR and FC/CB_50-60_ showed modest increasing trends but also high variability. In APPswe-PS1dE9 mice, DVR and FC/CB_50-60_ did not increase with age. Thioflavin S and anti-Aβ_1−40_ positive Aβ deposits were present in all transgenic mice at 19 to 22 months, and they co-localized with [^**18**^F]flutemetamol binding in the brain slices examined with *in vitro* and *ex vivo* autoradiography*.*

**Conclusions:**

Increased [^**18**^F]flutemetamol retention in the brain was detected in old APP23 mice *in vivo*. However, the high specific activity of [^**18**^F]flutemetamol did not provide a notable advantage in Tg2576 and APPswe-PS1dE9 mice compared to the previously evaluated structural analogue [^11^C]PIB. For its practical benefits, [^**18**^F]flutemetamol imaging with a suitable mouse model like APP23 is an attractive alternative.

## Background

Alzheimer's disease (AD) is the most common cause of dementia. The main pathological findings in a typical AD brain are neurofibrillary tangles and extracellular neuritic plaques, mainly composed of a fibrillar form of beta-amyloid (Aβ) peptide [1]. Methods based on molecular imaging have recently made it possible to assess brain Aβ deposition in patients with AD. Positron emission tomography (PET) with the Aβ imaging agent [^11^C]Pittsburgh compound B ([^11^C]PIB) first established this as a valuable biomarker approach for evaluating changes in Aβ deposition [2],[3]. Several small animal PET imaging studies have also been performed with [^11^C]PIB, but the results have not been consistent, probably reflecting differences in specific activity of the tracer and in the employed animal models [4]-[8]. Due to the short half-life (*T*_½_ = 20.4 min) of ^11^C, ^**18**^F-labelled ligands for Aβ imaging have been developed to extend the use of amyloid PET to the centres without on-site radionuclide production and tracer synthesis capacity. Due to their practical benefits, the use of ^**18**^F-labelled tracers has also raised interest in small animal imaging [9]-[11].

[^**18**^F]flutemetamol (2-(3-[^**18**^F]fluoro-4-(methylamino)phenyl)-1,3-benzothiazol-6-ol, [^**18**^F]3′F-PiB, [^**18**^F]GE067) is a ^**18**^F-labelled analogue of [^11^C]PIB developed and marketed by GE Healthcare (Buckinghamshire, UK). [^**18**^F]flutemetamol PET has been shown to successfully differentiate between patients with AD and healthy control subjects and to perform similarly to [^11^C]PIB in this respect [12],[13]. [^**18**^F]flutemetamol has shown high test-retest reliability and high specificity (96%, [12]) and sensitivity (93%, [12]) in the detection of AD, and regional imaging results obtained with it were consistent with AD plaque pathology in cortical biopsy samples [14]-[16]. Based on these findings, [^**18**^F]flutemetamol received FDA approval as a diagnostic agent for the assessments of Aβ deposition in the brains of adults evaluated for AD (FDA application number (NDA) 203137, GE Healthcare).

The present demonstration of principle study evaluated the applicability of [^**18**^F]flutemetamol to detect and quantitate the changes in brain Aβ deposition in APP23, Tg2576 and APPswe-PS1dE9 transgenic (TG) mouse models of AD. We expected that the very high specific activity of [^**18**^F]flutemetamol (>1 TBq/μmol) and thus lower injected mass to transgenic animals would be beneficial in small animal Aβ imaging. We also expected physical characteristics and practical benefits of the ^**18**^F-radionuclide to make [^**18**^F]flutemetamol an attractive preclinical Aβ imaging agent for preclinical studies.

## Methods

### Synthesis of [^18^F]flutemetamol

[^**18**^F]flutemetamol was produced with a FASTLab® Synthesizer (GE Healthcare), following analogous procedures to those described in the patent, WO 2007/020400 A1 ‘Fluorination process of anilide derivatives and benzothiazole fluorinate derivatives as *in vivo* imaging agents’. The specific radioactivity of [^**18**^F]flutemetamol was >1 TBq/μmol at synthesis completion (*N* = 34). Radiochemical purity exceeded 92%.

### Animal models

All animal experiments were approved by the Animal Experiment Board of the Province of Southern Finland (permissions ESAVI-2010-04454/Ym-23 and ESAVI/3899/04.10.07/2013) and the animal care complied with the guidelines of the International Council of Laboratory Animal Science (ICLAS). For *in vivo* imaging, we used mice from three different TG mouse lines and their strain-matched wild-type (WT) control animals. APP23 mice (APPswe [17]; Novartis Pharma (Basel, Switzerland); *N* = 4 TG females, 2 TG males and 4 WT females) overexpress the human 751 isoform of amyloid precursor protein (APP), which contains the Swedish K670N/M671L double mutation (APPswe). These mice develop Aβ plaques with dense cores, similar to human plaques, vascular Aβ, and neuron loss in the hippocampal CA1 pyramidal cell layer [18]. Tg2576 mice (APPswe, B6; SJL-Tg(APPSWE)2576Kha [19]; Taconic Europe. Ejby, Denmark; *N* = 4 TG females, 3 WT females) express the human 695 isoform of APPswe, which leads to expression of mutant Aβ, Aβ plaques and memory deficits. APPswe-PS1dE9 mice (B6C3-Tg(APPswe, PSEN1DE9)85Dbo/J [20]; Jackson Laboratories, Bar Harbor, ME, USA; *N* = 4 TG females, 3 TG males, 3 WT females) express a chimeric mouse/human 695 isoform of APPswe and mutant human Presenilin-1 (PS1dE9). The APPswe-PS1dE9 model mimics early-onset familial AD, with elevated Aβ_1-40_ and Aβ_1-42_ levels, abundant plaque pathology and memory deficits [21]. The APPswe-PS1dE9 male mice used in *ex vivo* studies were a kind gift from Professor H. Tanila from the University of Eastern Finland. The animals were group-housed at the Central Animal Laboratory of the University of Turku at controlled temperature (21 ± 3°C) and humidity (55 ± 15%) and with a light period from 6:00 a.m. to 6:00 p.m. TG and WT mice were fed with a soy-free diet (RM3 (E) soya, 801710, Special Diets Service, Essex, UK). The animals had free access to food and tap water (summary of animals, Table 1).

**Table 1:** Summary of the animals used for PET experiments

	**APP23**		**Tg2576**		**APPswe-PS1dE9**	
	**TG**	**WT**	**TG**	**WT**	**TG**	**WT**
All mice (*N*)	6	4	4	3	7	3
*In vivo* PET (*N*)						
9 mo	2	-	2	-	2	-
12 mo	1	-	4	2	3	2
15 mo	1	-	4	3	2	3
18 to 19 mo	2	-	2	2	2	3
21 to 22 mo	1	1	2	1	-	-
27 mo	-	1	-	-	-	-
*Ex vivo* ARG (*N*)						
18 to 19 mo	-	-	-	-	4	1
21 to 22 mo	-	-	1	1	-	-
24 to 27 mo	2	2	-	-	-	-

Amount of transgenic (TG) and wild-type (WT) animals used in this study. -, not available; ARG, autoradiography; mo, age in months.

### PET imaging

*In vivo* [^**18**^F]flutemetamol PET imaging was performed at 9, 12, 15, 18 to 19, and 21 to 22 months of age (in all 48 PET scans). At each time point, 1 to 4 TG and WT mice of each model were imaged (Table 1). Animals were anaesthetized with isoflurane, and computed tomography (CT) scans were acquired for attenuation correction and anatomical reference. Next, simultaneous with an i.v. injection of 4.3 ± 0.9 MBq (<5.5 ng, N = 48) of [^**18**^F]flutemetamol, a 60-min dynamic emission scan was started with an Inveon PET/CT device (Siemens Medical Solutions USA, Knoxville, TN, USA). Dynamic imaging data were divided into 51 imaging frames (30 × 10 s, 15 × 60 s, 4 × 300 s, 2 × 600 s); 3D imaging data were rebinned into 2D sinograms with a Fourier rebinning algorithm and then reconstructed with 2D filtered back-projection, with a final voxel size of 0.78 × 0.78 × 0.80 mm. Data were decay-corrected to the time of injection.

### Analysis of PET data

PET data were analysed with Inveon Research Workplace analysis software (Siemens Medical Solutions). PET and CT images were co-registered, and volumes of interest (VOI) were drawn under guidance of the CT image and a mouse brain atlas to cover the whole brain, the frontal cortex (FC) and the cerebellum (CB). Logan distribution volume ratios (DVR) were calculated from the dynamic PET data with the graphical method described by Logan et al. [22], with CB as the reference tissue and fit time from 5 to 60 min. FC/CB ratio curves were calculated for the entire 60-min scan. In addition, static FC/CB retention ratios were calculated only for the late phase (50 to 60 min) of the scan.

### *In vitro* binding experiment

*In vitro* binding experiments were performed as previously described [9] with 20-μm brain cryosections from transgenic APP23 (*N* = 2), Tg2576 (*N* = 2) and APPswe-PS1dE9 (*N* = 2) mice and wild-type control mice (*N* = 5) that were sacrificed after *in vivo* studies were finalized (Table 2). Briefly, the brain sections were incubated in 0.5 MBq/ml [^**18**^F]flutemetamol in 2% human serum albumin for 30 min. The specificity of [^**18**^F]flutemetamol binding was evaluated by adding 10 μM non-radioactive PIB (ABX, Radeberg, Germany) to the incubation solution, as previously described [9]. [^**18**^F]flutemetamol binding was detected using digital autoradiography (25 μm resolution).

**Table 2:** **Summary of the brain tissue used for** ***in vitro*** **experiments**

	**APP23**		**Tg2576**		**APPswe-PS1dE9**	
	**TG (mo)**	**WT (mo)**	**TG (mo)**	**WT (mo)**	**TG (mo)**	**WT (mo)**
*In vitro* ARG	18, 21	27	22	22	19	19
Aβ_1-40_	21, 27	24, 27	22	22	17, 19	19
Thioflavin S	21, 27	24, 27	22	22	17, 19	19

Ages of the transgenic (TG) and wild-type (WT) mice from which the brain cryosections for stainings in this study were obtained. ARG, autoradiography; mo, age in months.

### *Ex vivo* autoradiography

Mice were anaesthetized with isoflurane and intravenously injected with 9.1 ± 1.7 MBq (<7.5 ng, *N* = 11) of [^**18**^F]flutemetamol. After the tracer had distributed for 30 min, the mice were sacrificed by cardiac puncture. The brain was immediately dissected, rapidly frozen in chilled isopentane, and cut to 20-μm cryosections at the levels of the FC and CB. Sections were subsequently exposed to an imaging plate for approximately 4 h. Plates were read with a Fuji BAS-5000 analyser (resolution 25 μm). Regions of interest (ROI) were drawn onto the FC and CB with the Aida Image Analyzer analysis program (Aida 4.22, Raytest Isotopenmessgeräte GmbH, Straubenhardt, Germany). ROIs were drawn onto at least ten sections per animal, and mean intensity ratios were calculated between the FC and the CB. In addition, intensity profiles were drawn across the cortex that contained Aβ deposits, and Aβ plaque-to-background ratios were calculated for TG mice.

### *Ex vivo* biodistribution

We further investigated [^**18**^F]flutemetamol binding to different brain regions and structures inside the head in WT male C57Bl/6N mice (*N* = 4) using *ex vivo* tissue counting to better understand the observed binding *in vivo*. Mice anaesthetized with isoflurane were injected with 5.1 ± 0.9 MBq of [^**18**^F]flutemetamol, and the tracer was allowed to distribute for 60 min. Mice were sacrificed by cardiac puncture, and the brain was rapidly removed. We dissected the forebrain, midbrain, hindbrain, olfactory bulb, medulla, eyes, Harderian glands and salivary glands, and we collected samples of FC, CB, medulla and cranial bone. ^**18**^F-radioactivity was measured with a gamma counter (2480 WIZARD^2^, PerkinElmer, Turku, Finland). Measurements were decay-corrected, the background was subtracted and results were expressed as the percentage of injected dose per gram of tissue (% ID/g).

### Histological and immunohistochemical characterization of brain Aβ deposits

In the same cryosections subjected to *ex vivo* and *in vitro* autoradiography, fibrillar Aβ deposits were measured with the histochemical dye, Thioflavin S (ThS; Sigma-Aldrich, St. Louis, MO, USA), as previously reported [9]. Aβ pathology was also evaluated in adjacent tissue sections by immunohistochemical staining with an anti-Aβ_1-40_ (1:400; Millipore Corp., Billerica, MA, USA) antibody, as previously reported [4]. The mice used for brain histology are presented in Table 2. All sections were post-fixed in 4% paraformaldehyde for 30 min before staining. Fluorescent images were examined with a SteREO Lumar V.12 microscope (Carl Zeiss Microscopy GmbH, Jena, Germany), and the images were captured with a Zeiss Axiocam HRm S/N 1475 camera. Images of 3, 3′-diaminobenzidine stained sections were digitized with the Pannoramic 250 Flash II digital slide scanner (3DHistech, Budapest, Hungary), and the images were captured with the Pannoramic Viewer software.

### Data analysis and statistics

All mean values are expressed as mean ± SD. Results for less than three animals are given as individual values. Linear regression analysis was performed using GraphPad Prism software (version 5.01, La Jolla, CA, USA). *P* values < 0.05 were considered statistically significant.

## Results

### PET imaging

**Figure 1:**
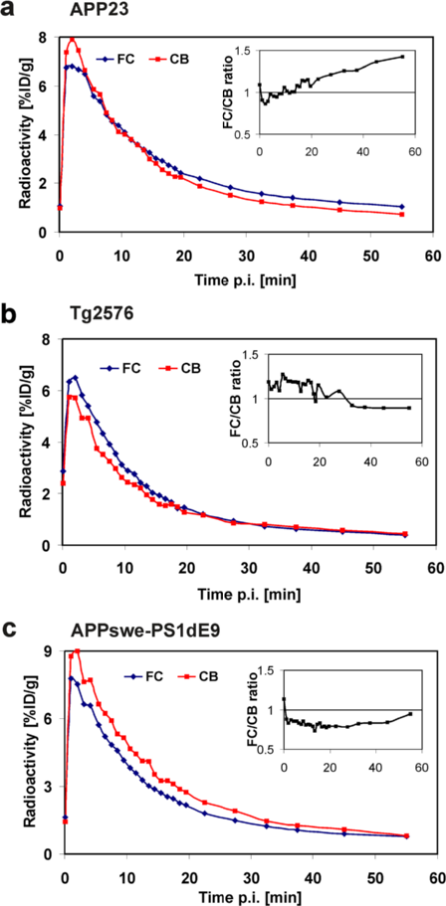
***In vivo*[**^**18**^**F]flutemetamol retention in the brain of mouse models of AD.** Sagittal PET/CT images summed over 40 to 60 min after [^**18**^F]flutemetamol injection are shown for **(a)** APP23, **(b)** Tg2576 and **(c)** APPswe-PS1dE9 mice. Increased [^**18**^F]flutemetamol retention (yellow) was detected in the cerebral cortex of aged APP23 mice (18 and 21 months), but signals in aged Tg2576 (19 and 22 months) and APPswe-PS1dE9 (19 months) mice were similar to those of the young (9 months) transgenic mice. White arrows, frontal cortex; red arrows, cerebellum; mo, age of animal in months.

**Figure 2:**
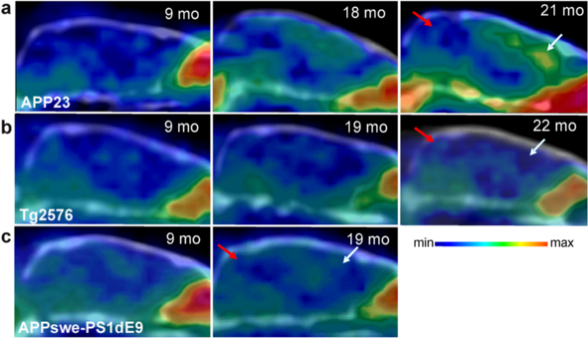
^**18**^**F-radioactivity uptake and washout in the brain of mouse models of AD.** Dynamic PET scans were performed for 60 min after [^**18**^F]flutemetamol injection, and presented time-radioactivity curves represent ^**18**^F-radioactivity uptake and washout in the frontal cortex (FC) and cerebellum (CB), expressed as percent of the injected dose (ID) per gram of brain tissue (%ID/g). Data are representative of **(a)** APP23 (21 months), **(b)** Tg2576 (22 months) and **(c)** APPswe-PS1dE9 (19 months) transgenic mice. (Insets) FC/CB ratios were calculated for 60 min dynamic scans. Only APP23 mice showed higher ^**18**^F-radioactivity in FC than in CB in the later time frames; thus, they had increasing FC-to-CB ratios.

Increased [^**18**^F]flutemetamol retention compared to young TG animals and WT control animals was observed in the FC of aged APP23 mice in visual assessment of the PET images (Figure 1a), but not in aged Tg2576 or APPswe-PS1dE9 mice (Figure 1b,c). Time-radioactivity curves in Figure 2 illustrate the time-course of [^**18**^F]flutemetamol uptake and washout in the FC and CB of representative TG animals at the final PET evaluation (at 19 to 22 months of age). The FC/CB ratios clearly increased over time in old APP23 mice. On the contrary, in Tg2576 and APPswe-PS1dE9 mice, the ratio was less than 1 in the later time frames.

**Figure 3:**
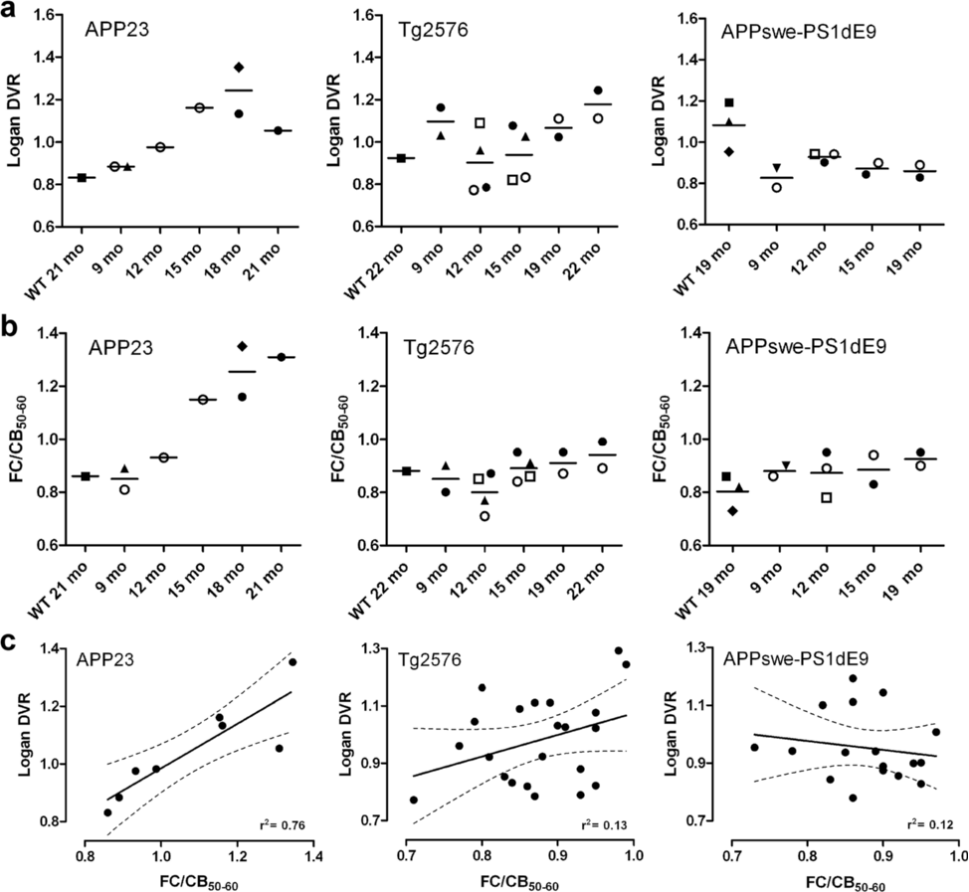
**Quantitation of [**^**18**^**F]flutemetamol binding in the brain of mouse models of AD. (a)** Logan distribution volume ratios (DVR) and **(b)** frontal cortex-to-cerebellum ratios from ^**18**^F-radioactivity data measured over 50 to 60 min (FC/CB_50-60_) were calculated for APP23, Tg2576 and APPswe-PS1dE9 transgenic mice, at different ages. For wild-type (WT) control mice, only imaging data from the last PET evaluation are shown for clarity. Different symbols indicate different individual mice. **(c)** DVRs and FC/CB_50-60_. Ratios correlated in APP23 mice but not in Tg2576 and APPswe-PS1dE9 mice (linear regression, *r*^2^ = 0.76, *p* = 0.0049, *N* = 9 (APP23); *r*^2^ = 0.13, *p* = 0.1046, *N* = 22 (Tg2576); *r*^2^ = 0.03, *p* = 0.5256, *N* = 17 (APPswe-PS1dE9)).

Logan DVRs calculated for the FCs of all TG and WT animals are presented in Figure 3a. Each individual mouse has its own symbol. The DVRs were increased in the aged APP23 TG mice compared to young TG and WT control animals. In younger TG mice, DVRs were 0.88 and 0.88 (9 months, *N* = 2) and 0.98 (12 months, *N* = 1). In older TG mice, DVRs were 1.16 (15 months, *N* = 1), 1.13 and 1.35 (18 months, *N* = 2), and 1.05 (21 months, *N* = 1). DVRs for WT animals were 0.83 (21 months, *N* = 1) and 0.98 (27 months, *N* = 1). The DVRs of the Tg2576 mice showed an increasing trend, reaching 1.24 and 1.11 (*N* = 2) at 22 months (WT 0.92, *N* = 1). In contrast, the APPswe-PS1dE9 mice tended to have lower DVRs (0.82 and 0.89, *N* = 2) than WT mice (1.08 ± 0.12, *N* = 3) at 19 months.

FC/CB_50-60_ ratios calculated for the FCs of all TG and WT animals are presented in Figure 3b. Each individual mouse has its own symbol. The FC/CB_50-60_ ratios increased with age in APP23 mice. The FC/CB_50-60_ values were 0.81 and 0.89 (9 months, *N* = 2) and 0.93 (12 months, *N* = 1) in younger TG mice, increasing to 1.15 (15 months, *N* = 1), 1.16 and 1.35 (18 months, *N* = 2) and 1.31 (21 months, *N* = 1) in older TG mice. In strain-matched WT mice, FC/CB_50-60_ values were 0.86 (21 months, *N* = 1) and 0.99 (27 months, *N* = 1). In Tg2576 mice, FC/CB_50-60_ values were 0.80 ± 0.07 (*N* = 4) at 12 months (WT 0.81 and 0.79, *N* = 2) and 0.89 and 0.99 (*N* = 2) at 22 months (WT 0.88, *N* = 1). In APPswe-PS1dE9 mice, FC/CB_50-60_ remained almost stable from 0.87 ± 0.08 (*N* = 3) at 12 months (WT 0.85 and 0.90, *N* = 2) to 0.95 and 0.90 (*N* = 2) at 19 months (WT 0.80 ± 0.06, N = 3). The FC/CB_50-60_ ratios agreed well with the Logan DVR values in APP23 mice (*r*^2^ = 0.76, *p* = 0.0049; *N* = 9), but similar agreement was not seen in Tg2576 (*r*^2^ = 0.13, p = 0.1046; N = 22) or APPswe-PS1dE9 mice (r^2^ = 0.03, p = 0.5256; N = 17) (Figure 3c).

Some transgenic mice were imaged repeatedly at several time points; this is indicated by the different symbols of individual animals in Figure 3a,b. In APP23 mice, FC/CB_50-60_ clearly increased from 9 to 15 months and from 18 to 21 months. Also, DVR values showed clear increase from 9 to 18 months. However, one TG mouse showed lower DVR value at 21 months compared to 18 months. Some Tg2576 mice showed modest increases in FC/CB_50-60_. Among APPswe-PS1dE9 mice, no increases were observed in individual mice subjected to repeated PET scans.

### *In vitro* binding experiment

**Figure 4:**
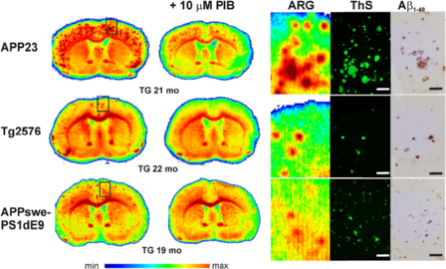
***In vitro*binding of [**^**18**^**F]flutemetamol to Aβ deposits in the brain of mouse models of AD.** Autoradiographs of brain sections from transgenic (TG) APP23 (21 months), Tg2576 (22 months) and APPswe-PS1dE9 (19 months) transgenic mice show [^**18**^F]flutemetamol binding to whole sections (left panels) and adjacent sections treated with 10 μM cold PIB (+10 μM PIB) (right panels). High magnifications of areas enclosed in boxes show Aβ deposits detected with [^**18**^F]flutemetamol autoradiography (ARG, left column), compared to the same section stained with Thioflavin S (ThS, middle column), and an adjacent section stained with an anti-Aβ antibody (Aβ_1-40_, right column). Scale bar = 200 μm. mo, age of animal in months.

In all brain sections from transgenic APP23, Tg2576 and APPswe-PS1dE9 mice incubated with [^**18**^F]flutemetamol *in vitro*, the bound radioactivity co-localized well with fibrillar Aβ plaques stained with ThS and anti-Aβ_1-40_ antibody (Figure 4). The punctate [^**18**^F]flutemetamol binding was totally blocked in Tg2576 and APPswe-PS1dE9 sections co-incubated with 10 μM cold PIB, but only partially blocked in APP23 sections, likely because of their very high plaque load (Figure 4).

### *Ex vivo* autoradiography

**Figure 5:**
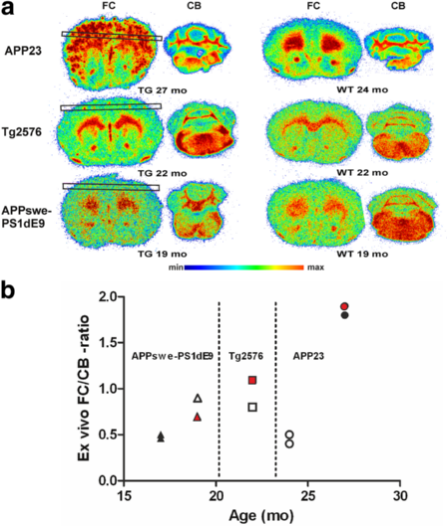
***Ex vivo*binding of [**^**18**^**F]flutemetamol to Aβ deposits in the brain of mouse models of AD. (a)** Representative brain sections are shown for transgenic (TG) APP23 (27 months), Tg2576 (22 months) and APPswe-PS1dE9 (19 months) mice and wild-type (WT) control mice. Plaque-to-background ratios were calculated from the intensity profiles derived from the areas enclosed in black boxes (*FC column*). **(b)** Frontal cortex (FC) to cerebellum (CB) ratios were calculated for TG (filled symbols) and WT (open symbols) animals at different ages. The red symbols represent the ratios from the individual TG animals presented in **(a)**. mo, age of animal in months.

Digital autoradiograms of the FC and CB were acquired from the brain sections of the three evaluated mouse models and wild-type control mice (Figure 5a). Intensity profiles (derived from the boxed regions in the FC images of Figure 5a) showed that Aβ plaques emitted higher signals than the surrounding cortex; the maximum Aβ plaque-to-background ratios were 3.2 and 2.6 for two APP23 mice, and 4.8 for a Tg2576 mouse. In four APPswe-PS1dE9 mice, [^**18**^F]flutemetamol binding to Aβ deposits in the FC was not visually equally obvious as in the APP23 and Tg2576 TG mice. The FC intensity profiles resembled those of WT control mice, and plaque-to-background ratios did not allow sharp plaque identification and were close to unity. The FC/CB ratios were clearly elevated for APP23 mice compared to WT mice (Figure 5b). The single investigated Tg2576 mouse also showed a higher FC/CB ratio than its WT control, but all APPswe-PS1dE9 TG mice showed lower FC/CB ratios than their WT control. High non-specific [^**18**^F]flutemetamol binding was observed in the cerebral and cerebellar white matter in all evaluated TG and WT mice (Figure 5a).

### *Ex vivo* biodistribution

^**18**^F-radioactivity uptake in the brain and intracranial structures of WT C57Bl/6N animals are shown in Table 3. The highest ^**18**^F-radioactivity in the brain was measured in the medulla and hindbrain. ^**18**^F-radioactivity in the CB was higher than that in the FC. The highest ^**18**^F-radioactivity was measured from the structures inside the nasal cavity.

**Table 3:** ***Ex vivo*biodistribution of [**^**18**^F**]flutemetamol in the mouse head**

	**Mean**	**SD**
Brain areas		
Brain	0.21	0.05
Forebrain	0.19	0.05
Midbrain	0.24	0.04
Hindbrain	0.29	0.07
Olfactory bulb	0.18	0.03
Cortex	0.14	0.02
Cerebellum	0.19	0.06
Medulla	0.86	0.20
Extracranial tissue		
Blood	0.32	0.01
Plasma	0.52	0.02
Erythrocytes	0.12	0.01
Cranial bone	0.83	0.13
Submandibular salivary gland	0.16	0.02
Sublingual gland	0.28	0.03
Harderian gland	0.36	0.05
Eyes	0.93	0.11
Nasal cavity	1.89	0.47

Uptake of [^**18**^F]flutemetamol in the brain and adjacent tissues of C57Bl/6 N mice at 60 min post-injection of the tracer (*N* = 4). Values are presented as percentage of injected dose per gram of tissue (% ID/g).

### Aβ deposition in the brain

**Figure 6:**
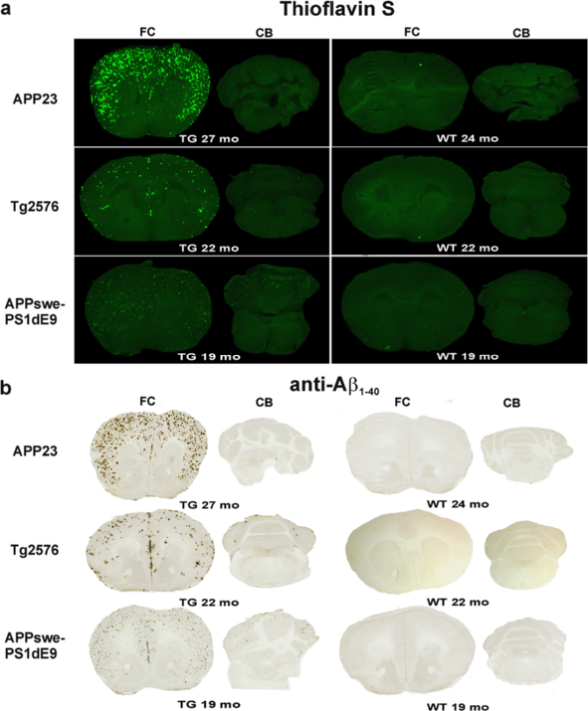
**Aβ deposition in the brain of mouse models of AD. (a)** Representative images show Thioflavin S staining for transgenic (TG) APP23 (27 months), Tg2576 (22 months) and APPswe-PS1dE9 (19 months) mice and wild-type (WT) control mice in the same sections used for *ex vivo* evaluation of [^**18**^F]flutemetamol binding in Figure 5. **(b)** Adjacent sections from TG APP23 (27 months), Tg2576 (22 months) and APPswe-PS1dE9 (19 months) mice and WT control mice were stained with specific anti-Aβ_1-40_ antibody mo, age of animal in months.

The same brain sections investigated with *ex vivo* and *in vitro* autoradiography were stained with ThS and adjacent sections with an anti-Aβ_1-40_ antibody (Figures 4 and 6) to determine the presence and localization of Aβ deposits in the brain and their co-localization with [^**18**^F]flutemetamol binding. In APP23 brains at 21 and 27 months, large, spherical Aβ deposits with a dense core were seen in the FC and in other forebrain regions, but not in the CB. Deposits were prominently stained with ThS and anti-Aβ_1-40_. The plaque load was modest in Tg2576 brain at 22 months, but the deposits were stained with ThS and anti-Aβ_1-40_ antibody and the staining co-localized with [^**18**^F]flutemetamol binding in the FC. In APPswe-PS1dE9 mice, Aβ deposits were small, spherical and showed low intensity staining with ThS at 19 months. However, deposition was abundant throughout the brain, including the CB, at 19 months. WT mice did not have detectable Aβ deposits (Figure 6).

## Discussion

In the present study, we evaluated the applicability of [^**18**^F]flutemetamol to assess brain Aβ deposition in small animal *in vivo* PET imaging. [^**18**^F]Flutemetamol has already been extensively investigated in humans and has received marketing approval as a radiopharmaceutical for the detection of Aβ deposition in subjects with possible AD [12],[23]-[25]. In our previous animal study, we found that the pharmacokinetic properties of [^**18**^F]flutemetamol in WT rats and mice were suitable for preclinical imaging, and [^**18**^F]flutemetamol was found to bind to Aβ deposits in the Tg2576 mouse brain, both *in vitro* and *ex vivo*[9]*.* To our knowledge, the present demonstration of principle study is the first to show that it is possible to detect increase in [^**18**^F]flutemetamol retention *in vivo* in the mouse brain parallel with increasing age. However, in the present study, increased [^**18**^F]flutemetamol retention was evident only in the transgenic APP23 mice and not in Tg2576 or APPswe-PS1dE9 mice. APP23 mouse model is known to have relatively slow plaque deposition and large Aβ deposits with dense amyloid cores [4],[18]. Tg2576 mice have late onset and even slower rate of Aβ deposition than APP23 mice [4]. In APPswe-PS1dE9 mice, small Aβ deposits are present already at earlier age, and deposition is abundant throughout the brain in older animals [4].

Varying results have been found in several small animal imaging studies with [^11^C]PIB, a structural analogue of [^**18**^F]flutemetamol which is still considered the golden standard of brain amyloid imaging. A possible explanation for the variation is that different TG mice are likely to express different Aβ isoforms with different amounts of high-affinity binding sites for amyloid tracers [26]. We hypothesized that very high specific activity and lower injected mass of [^**18**^F]flutemetamol might provide an advantage in some animal models that did not show increased specific binding with [^11^C]PIB in our previous study, perhaps because of low levels of high-affinity binding sites [4]. In this study, the injected mass of [^**18**^F]flutemetamol was less than 5.5 ng in all *in vivo* cases (*N* = 48). This is even at its worst ten times less than previously reported with [^11^C]PIB (68 ± 23 ng; *N* = 50 [4]). However, the results revealed that the use of a tracer with high specific radioactivity did not as such provide any advantage in PET imaging of APPswe-PS1dE9 mice *in vivo*. In APPswe-PS1dE9 mice, plaque deposition was abundant throughout the brain (including the CB) at 19 months, but plaque composition appeared to be different from the other TG models, with only small fibrillar deposits that could bind ThS and related Aβ imaging agents. In Tg2576 mice, modest increases in DVR and FC/CB_50-60_ ratio were seen as the individual transgenic mice aged. In a previous study with [^11^C]PIB and Tg2576 mice, no such trend was seen [4]. In addition, [^**18**^F]flutemetamol did bind to Aβ in the cortical sections from Tg2576 animals, both *ex vivo* and *in vitro*, consistent with previous *ex vivo* results [9]. However, the amount of plaques was modest even at 22-month-old animals, explaining the modest increases in *in vivo* binding ratios. We concluded that Tg2576 and APPswe-PS1dE9 mouse models are not suited to small animal imaging studies with tracers that shared binding sites with [^11^C]PIB and [^**18**^F]flutemetamol, due to their plaque structure or low plaque load.

Our study is difficult to compare with previous small animal amyloid imaging PET studies that used other ^**18**^F-labelled tracers, due to the differences in tracers, quantitation methods and animal models used. One previous study did not observe increased cortical tracer retention with [^**18**^F]FDDNP in 13 to 15-month-old Tg2576 mice [27]. The authors concluded that specific [^**18**^F]FDDNP binding was insufficient and that insufficient spatial resolution and partial volume effects (PVE) limited the precision of measurements in small VOIs [27]. However, in a more recent study with [^**18**^F]florbetaben, Aβ deposition was longitudinally followed in the APPswe mouse model from age 13 to 20 months, and the results were consistent with our results with [^**18**^F]flutemetamol in APP23 mice. In that study, the PET results agreed well with histopathological brain Aβ, and differentiation was further improved when a PVE correction was applied [10],[28]. Using the APP-PS1-21 mouse model, also specific binding of [^**18**^F]florbetapir was shown to increase from age 3 to 8 months; however, no further increase was detected at 12 months compared to the baseline scan at 3 months [11]. At the end of synthesis, the specific activities of [^**18**^F]florbetaben (80 GBq/μmol, [10]) and [^**18**^F]florbetapir (150 to 220 GBq/μmol, [11]) were much lower than the specific activity of [^**18**^F]flutemetamol in this study (>1 TBq/μmol). This difference further supports the higher importance of the chosen mouse model over high specific activity of the tracer for successful imaging results.

Similarly to human studies, the CB has usually been used as a reference region in small animal amyloid imaging studies. However, several limitations should be considered when applying this approach. In the mouse brain, the white and grey matter cannot be distinguished when the reference region VOIs are drawn over the CB, due to its small size. As a result, because all ^**18**^F-labelled β-amyloid imaging tracers typically exhibit prominent white matter binding, the concentration of radioactivity in the CB can be higher than that in the cortical grey matter ROIs. In our study, DVRs were less than unity in WT animals and in young TG animals, which presumably had only modest plaque loads. Negative BP_ND_ values for WT and young TG mice were also previously reported for [^**18**^F]florbetaben. Only TG mice that were 16 and 20 months old showed positive BP_ND_s (0.04 ± 0.07 and 0.10 ± 0.11, respectively) [10]. For small animal imaging, tracers with low non-specific binding to white matter would be beneficial, such as the novel ^**18**^F-labelled fluoropyridyl derivative, BAY 1008472, or the benzofuran ligand [^**18**^F]AZD4694 [29],[30]. Moreover, for APPswe-PS1dE9 mice, the CB is not a suitable reference region because Aβ deposits are present at 19 months of age.

Imaging of small structures in the mouse brain, of a size close to the spatial resolution of the PET scanner, will inevitably be affected by PVEs; thus, measurable signals will be less precise than those achievable with digital autoradiography. Consistent with previous [^**18**^F]florbetaben and [^**18**^F]florbetapir studies, the FC/CB ratios observed in our study with [^**18**^F]flutemetamol with *ex vivo* autoradiography (1.8 and 1.9, respectively, at 30 min p.i.) were higher than that observed with *in vivo* PET (1.3 at 30 to 35 min p.i.) for old APP23 mice. Adding PVE correction could further improve quantitation of [^**18**^F]flutemetamol PET data, especially in animals with still only modest plaque load. Also, spillover from tissues close to the brain could introduce errors to the VOI-based analysis method in mice. We observed high radioactivity in close proximity to the brain *in vivo*, and subsequent *ex vivo* tissue counting experiments confirmed that significant radioactivity was located outside of the brain, inside the nasal cavity. In addition, ^**18**^F-radioactivity concentrations detected in the mouse cranial bone (0.83 ± 0.13% ID/g at 30 min) were higher than those previously reported in rats (0.04 ± 0.01% ID/g at 30 min [9]); this suggests that more [^**18**^F]flutemetamol defluorination could be taking place in mice than in rats, reflecting more active metabolism. However, spillover from these structures would presumably lead to similar overestimation in the brains of all three TG mouse models.

One limitation of the present study was the small number of transgenic animals, especially the ones that could be imaged repeatedly over multiple time points. However, we wanted to evaluate the applicability of [^**18**^F]flutemetamol PET to small animal imaging in three different transgenic models even with limited statistical power, rather than in only one transgenic model with higher sample size and better statistical power. This demonstration of principle type of study was still able to show clear increasing trend in [^**18**^F]flutemetamol binding in aging APP23 mice and the lack of binding in Tg2576 and APPswe-PS1dE9 mice. Another limitation was that we could not perform *ex vivo* studies for all of the female mice imaged *in vivo*; however, the brain sections from these mice were used for *in vitro* binding experiments, and additional *ex vivo* studies were performed later, with aged males. Male APP23 mice were reported to show slightly slower deposition of Aβ plaques compared to females [17], but these mice were 27 months old; thus, Aβ deposition was abundant. In addition, a hybrid PET/MRI scanner with better spatial resolution and MR details of the brain would better serve the purposes of similar experiments.

## Conclusions

*In vivo* small animal imaging of brain Aβ with [^**18**^F]flutemetamol, similarly to its structural analogue [^11^C]PIB, is more dependent on which AD mouse model is used than its high specific activity. In this study, Aβ deposition in the brain could only be followed *in vivo* in APP23 mice. However, for its practical benefits, such as more efficient use of the produced tracer batches, [^**18**^F] flutemetamol imaging with a suitable mouse model like APP23 is an attractive alternative.

## Abbreviations

AD: Alzheimer's disease

Aβ: beta-amyloid

FC: frontal cortex

DVR: distribution volume ratio

CB: cerebellum

[^11^C]PIB: [^11^C]Pittsburgh compound B

PET: positron emission tomography

TG: transgenic

WT: wild-type

APP: amyloid precursor protein

CT: computed tomography

VOI: volume of interest

ROI: region of interest

ThS: Thioflavin S

## Competing interests

The authors declare that they have no competing interests.

## Authors' contributions

AS contributed to the design of the study, acquired, analysed and interpreted the PET data and histological and immunohistochemical stainings. AS drafted the manuscript. JR participated in the design of the study and carried out *in vitro* PET experiments. FL participated in the immunohistochemical stainings and performance of PET experiments. OE performed the radiochemical synthesis of [^**18**^F]flutemetamol. MS and GF participated in the design of the animal studies and revised the manuscript critically. MS drafted and revised the manuscript critically for its intellectual content. OS, JR and MHS contributed to the conception, design and coordination of the study, revised the manuscript critically for its intellectual content and gave final approval for the published version. MHS helped to draft the manuscript. All authors read and approved the final manuscript.
